# An ontology-based exploration of the concepts and relationships in the activities and participation component of the international classification of functioning, disability and health

**DOI:** 10.1186/2041-1480-3-1

**Published:** 2012-02-28

**Authors:** Vincenzo Della Mea, Andrea Simoncello

**Affiliations:** 1 Medical Informatics, Telemedicine and eHealth Lab, Dept. of Mathematics and Computer Science, University of Udine, Italy; 2 Friuli - Venezia Giulia Regional Central Health Directorate, Classification Area, WHO-FIC Collaborating Centre, Udine, Italy

## Abstract

**Background:**

The International Classification of Functioning, Disability and Health (ICF) is a classification of health and health-related issues, aimed at describing and measuring health and disability at both individual and population levels. Here we discuss a preliminary qualitative and quantitative analysis of the relationships used in the Activities and Participation component of ICF, and a preliminary mapping to SUMO (Suggested Upper Merged Ontology) concepts. The aim of the analysis is to identify potential logical problems within this component of ICF, and to understand whether activities and participation might be defined more formally than in the current version of ICF.

**Results:**

In the relationship analysis, we used four predicates among those available in SUMO for processes (Patient, Instrument, Agent, and subProcess). While at the top level subsumption was used in most cases (90%), at the lower levels the percentage of other relationships rose to 41%. Chapters were heterogeneous in the relationships used and some of the leaves of the tree seemed to represent properties or parts of the parent concept rather than subclasses. Mapping of ICF to SUMO proved partially feasible, with the activity concepts being mapped mostly (but not totally) under the IntentionalProcess concept in SUMO. On the other hand, the participation concept has not been mapped to any upper level concept.

**Conclusions:**

Our analysis of the relationships within ICF revealed issues related to confusion between classes and their properties, incorrect classifications, and overemphasis on subsumption, confirming what already observed by other researchers. However, it also suggested some properties for Activities that could be included in a more formal model: number of agents involved, the instrument used to carry out the activity, the object of the activity, complexity of the task, and an enumeration of relevant subtasks.

## Background

Terminologies and classifications are particularly crucial in the medical field, as they are widely diffused to represent knowledge in the clinical practice. Perhaps the most known terminologies and classifications are the International Classification of Diseases (ICD) [1] and the Systematized Nomenclature of Medicine (SNOMED) [2].

Ontologies are means for specifying the intended meaning of a vocabulary in a logical manner [3]. Depending on generality, we can distinguish between upper (or top-level) and domain ontologies. The former describe very general concepts like space, time, matter, object, event, action, etc., which are independent of a particular problem or domain. The latter describe the vocabulary related to a generic domain or a generic task or activity, by specializing the terms of an upper ontology.

The International Classification of Functioning, Disability and Health (ICF) [4,5] is a classification of health and health-related issues. ICF is WHO's framework for describing and measuring health and disability at both individual and population levels, and has been adopted by WHO Member States as a standard. ICF is freely available at http://apps.who.int/classifications/icfbrowser/.

**Figure 1:**
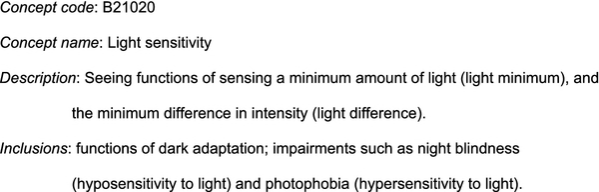
**A sample concept from the ICF Body functions component**.

ICF is a classification with a simple structure. It is divided into four main components, independent of each other: Body Functions, Body Structure, Activities and Participation, and Environmental Factors. A hierarchical structure is present, where each concept has a name, a text description, and inclusion and exclusion relationships. The first level of hierarchy is constituted of chapters. Each concept at each level has a hierarchical alphanumeric code. Figure 1 shows an example of concept from the Body Functions component.

Moreover, in its practical use, each concept is qualified by a numerical value, the interpretation of which is defined within a range specific for every chapter. However, this feature will not be considered in our analysis.

WHO develops and updates a family of health care terminologies including ICF, and has embarked on an open web-based cooperation to revise ICD 11 using ontology-driven tools [6]. The same process has been envisaged for future versions of ICF.

As reported in [7], a thorough analysis reveals that at least some components of ICF exhibits non-conformance to many formal ontological principles. Kumar and Smith concentrated on the Body Functions component of ICF and pointed out seven main ontology-related shortcomings of ICF classification [7].

Maintenance and evolution of ICF are among the tasks carried out by the WHO Family of International Classification Network, which is composed of 14 collaborating centers established at some national level institutions in the world. This work has been carried out among the activities of the Italian Center, as a basis for the development of a more formal version of ICF, in cooperation with other WHO-FIC collaborating centers.

In this paper, we present an ontology-based exploration of the relationships and concepts used in the Activities and Participation component of ICF, by mapping it to an upper ontology (specifically, SUMO: Suggested Upper Merged Ontology [8]). The aim of the analysis is to identify potential logical problems within this component of ICF and to understand whether activities and participation might be better defined in order to encompass properties and qualities described in form of subclasses in the current version of ICF. This work contributes to the recognition of the current status of ICF concepts related to activities and participation from an ontological point of view.

## Methods

The Activities and Participation component describes concepts defined as follows [4]:

*Activity is the execution of a task or action by an individual. Participation is involvement in a life situation*.

The Activities and Participation component is composed of nine chapters (Table 1). Each chapter has its own hierarchy; some chapters have a first level made of blocks, i.e., groupings of homogenous and consecutive categories (e.g., *d450-d469: Walking and moving*), while other chapters are directly organised into categories, i.e., three digit codes. Categories may have further subcategories. In total, there are 118 categories (partially grouped into 18 blocks) and 266 subcategories.

**Table 1:** Definitions of the nine chapters composing the Activities and Participation component of ICF

Chapter	Term	Definition
**d1**	Learning and applying knowledge	learning, applying the knowledge that is learned, thinking, solving problems, and making decisions.
**d2**	General tasks and demands	general aspects of carrying out single or multiple tasks, organizing routines and handling stress. These items can be used in conjunction with more specific tasks or actions to identify the underlying features of the execution of tasks under different circumstances.
**d3**	Communication	general and specific features of communicating by language, signs and symbols, including receiving and producing messages, carrying on conversations, and using communication devices and techniques.
**d4**	Mobility	moving by changing body position or location or by transferring from one place to another, by carrying, moving or manipulating objects, by walking, running or climbing, and by using various forms of transportation.
**d5**	Self-care	caring for oneself, washing and drying oneself, caring for one's body and body parts, dressing, eating and drinking, and looking after one's health.
**d6**	Domestic life	carrying out domestic and everyday actions and tasks. Areas of domestic life include, caring for one's belongings and space, acquiring food, clothing and other necessities, household cleaning and repairing, caring for personal and other household objects, and assisting others.
**d7**	Interpersonal interactions and relationships	carrying out the actions and tasks required for basic and complex interactions with people (strangers, friends, relatives, family members and lovers) in a contextually and socially appropriate manner.
**d8**	Major life areas	carrying out the tasks and actions required to engage in education, work and employment and to conduct economic transactions.
**d9**	Community, social and civic life	actions and tasks required to engage in organized social life outside the family, in community, social and civic areas of life.

After a first examination of the main hierarchy of the component, we analysed together all the relationships between parent and child concepts to understand i) whether subsumption is the most appropriate relationship and, if not, which is the specific relationship; and ii) whether the current relationship could link the parent concept to a property of it rather than to a subclass. The latter step aimed at pointing out the process properties that have been considered relevant by the developers of ICF to describe human functioning and health. Particular attention was given to the lower levels of the hierarchy. The specific relationships were then chosen, when possible, among those available in SUMO.

In our analysis, residual classes (e.g., "Other specified" and "Unspecified") were not included.

To graphically document the analysis, the Freemind mindmapping software [9] was used.

In order to better understand whether the concepts described among ICF activities and participation are uniform or rather include different logical kinds, we attempted a mapping towards an upper level ontology. Among the available upper ontologies we chose SUMO (Suggested Upper Merged Ontology) [8], because it has been mapped to the Wordnet dictionary [10], helping thus in the retrieval of concepts. It was developed within the IEEE Standard Upper Ontology Working Group as a possible standard upper ontology.

We converted the ClaML [11] representation of ICF into OWL [12], and then loaded it into Protégé [13], together with an OWL representation of SUMO. ICF concepts were then searched using Wordnet [14], from which, when possible, candidates for the mapping to SUMO were obtained. We manually carried out the mapping by searching ICF concepts first directly in SUMO and, when not found, in Wordnet. ICF concepts that according to our relationship analysis represented properties of the parent concept were not mapped.

In our analysis, we chose to use the following four predicates among those available in SUMO for processes: Patient, Instrument, Agent, subProcess.

The *Patient *predicate was adopted whenever a child concept in ICF was a subclass of the parent concept, where the specific feature of the subclass is given by the object involved in the activity. The *Instrument *predicate was adopted when a child concept differed from its sibling for the tool/means used to carry out the activity. The *Agent *predicate was used when a child concept differed from its siblings for a different number of agents involved (typically one, two or more). The *subProcess *predicate was adopted to identify child concepts related to different stages of the parent concept.

While we could have used the subsumption relationship in any other case, we decided to adopt another property that we were not able to identify in SUMO to describe complexity of activities. In fact, in some classes there is a distinction between "simple" and "complex" version of the activity. As this is a sensible difference from the user point of view, we maintained such a distinction, even if the definition of "simple" and "complex" is not provided.

## Results and discussion

The Activities and Participation component embodies two different concepts, namely activity and participation. ICF experts consider activity and participation as two facets of the same involvement of people in areas of life, respectively from individual and societal points of view. The ICF activity can be considered an intentional process according to SUMO. Participation is more difficult to define, although it seems to be related to a role in a scenario.

When looking at the first level of the hierarchy, two cases of heterogeneity become apparent.

**Figure 2:**
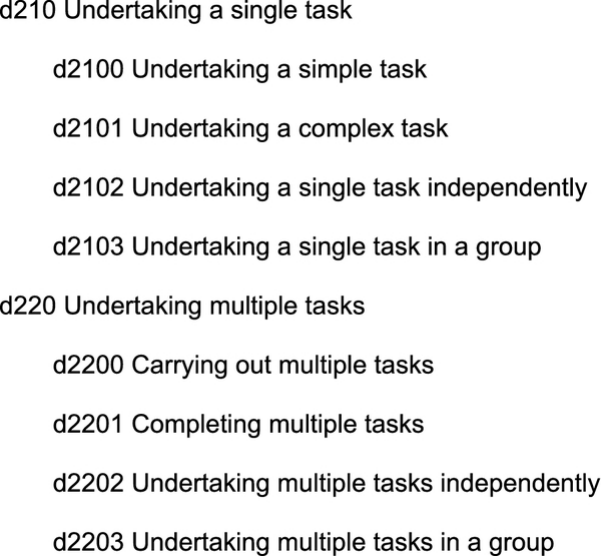
**Part of the Chapter 2 hierarchy (General tasks and demands)**.

In fact, Chapter 2 (General tasks and demands) differs from the other chapters, because it provides a sort of generic classification of tasks based on complexity and other features. Unfortunately, this classification does not provide explicit linkages to the concrete activities described in the rest of the Activities and Participation component, except for examples provided in the textual description. As an example, Figure 2 shows a part of Chapter 2 hierarchy.

Similarly, Chapter 7 (Interpersonal interactions and relationships) is a metamodel for social interaction.

### Analysis of relationships

A quantitative summary of the results of the analysis is shown in Table 2, where the relationships at the top levels (from chapter to categories) are described separately from those at the lower levels (i.e., from categories to their subclasses). A graphical representation is provided as additional material (see Additional file 1). While at the top level subsumption was used in most cases (90%), at the lower levels the percentage of other relationships rose to 41%. In four chapters (d1, d2, d3, d7), there was a majority of relationships more adequate than subsumption (100%, 93%, 100% and 56%, respectively). One chapter (d9) fully relied on the subsumption relationship.

**Table 2:** Relationships used in the ICF Activities and Participation component.

	Number of relationships						
**Chapter**	***total***	***Subsumption***	***subProcess***	***Agent***	***Patient***	***Instrument***	***Complexity***

d1	19/4	19/0	0/0	0/0	0/0	0/0	0/4
d2	3/14	1/1	2/4	0/4	0/3	0/0	0/2
d3	14/16	14/0	0/3	0/4	0/8	0/1	0/0
d4	17/50	17/33	0/0	0/0	0/6	0/4	0/7
d5	7/19	7/17	0/0	0/0	0/2	0/0	0/0
d6	9/26	9/24	0/0	0/0	0/0	0/0	0/2
d7	9/27	3/12	0/2	0/0	4/12	0/1	2/0
d8	15/8	13/5	0/3	0/0	0/0	0/0	2/0
d9	5/11	5/11	0/0	0/0	0/0	0/0	0/0
Total	**98/175**	**88/103**	**2/12**	**0/8**	**4/31**	**0/6**	**4/15**

The first number in each cell describes how many relationships of a specific kind are used in top level relationships; the second number describes how many relationships are used in subclasses of categories (i.e., relative to four and five digit codes)

**Figure 3:**
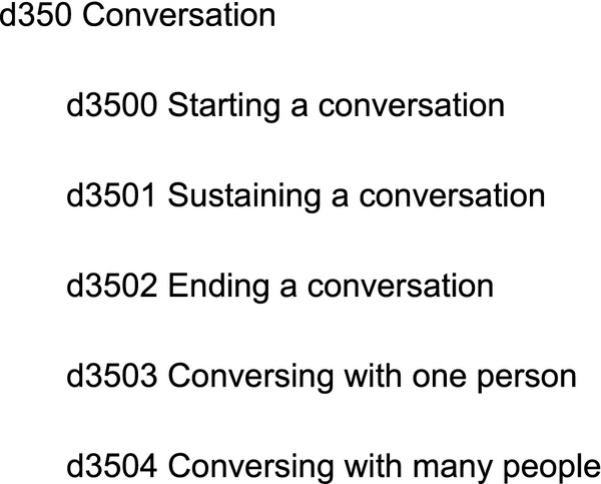
**The *Conversation *concept in ICF and its children concepts**.

Chapters were heterogeneous in the relationships used and some of the leaves of the tree seemed to represent properties or parts of the parent concept rather than subclasses. The subtree in Figure 3 clearly shows this: the first three children specify a subprocess of the *Conversation *activity, the latter two the number of agents involved.

Different chapters seemed to exhibit different patterns of relationships used. The number of involved agents appears as a relationship only in chapters 2 and 3. As already mentioned, Chapter 2 is an exception because it provides a classification of generic activities. Chapter 3 is devoted to communication, so there is a need for coding the number of people involved in communication (e.g. *d3550: Discussion with one person*, *d3551: Discussion with many people*).

Patient and Complexity properties are more widely spread, although it should be noted that Complexity has been used to code a variety of situations where the focus of ICF appears to be on the difficulty of the task.

### Mapping to SUMO

**Figure 4:**
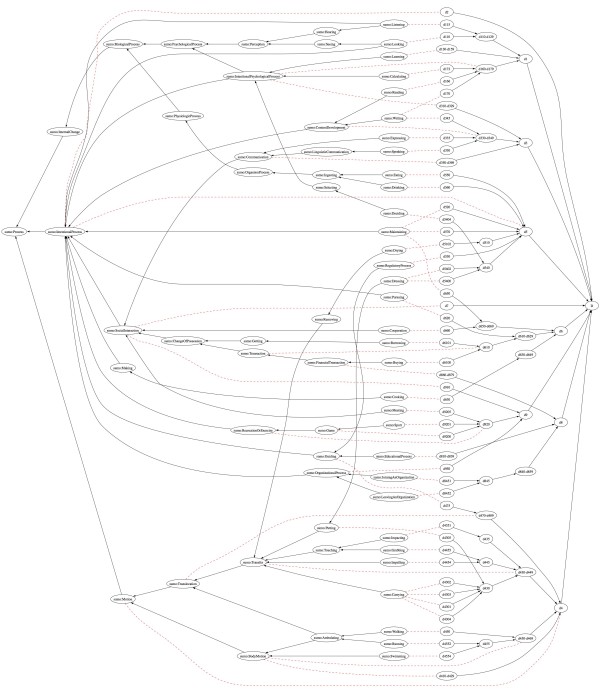
**ICF to SUMO mapping**.

The attempt to map ICF to SUMO, restricted to the Activities and Participation component, allowed to link ICF concepts to similar, usually broader, concepts from SUMO. Most concepts were mapped to subclasses of the Process concept. We were able to map 4 chapters (out of 9), 11 blocks (out of 18), 22 categories (out of 118) and 21 subcategories (out of 266). Figure 4 shows the mapping.

Chapter 1 is about learning and applying knowledge. When trying to map specific ICF concepts, only few of them find a similar target in SUMO. For example, the last block of the chapter, *Applying knowledge (d160-d179)*, has some subclasses recognizable in SUMO (e.g., *d170 Writing*, *d172 Calculating*), but other classes are not (e.g., *d175 Solving problems*), thus it can be overall mapped to *IntentionalPsychologycalProcess*.

Chapter 2 (*General tasks and demands*) may be generally considered a subclass of *IntentionalProcess*, without more specific mappings. For this reason, Chapter 2 could become the basis of a domain ontology on Activities, by providing classes to which to classify other activities, including those described in the next chapters.

Chapter 3 deals with communication, which according to ICF has a recipient role (*d310-d329 Communicating - Receiving*), a sender role (*d330-d349 Communicating - Producing*), and a conversation part inclusive of communication devices usage (*d350-d369 Conversation and use of communication devices and techniques*). Since communication in SUMO is modelled in a slight different way, the conversation concept in ICF was mapped to SUMO *Communication*, whereas the sender side of communication was mapped (with some doubts) to *ContentDevelopment*. The recipient side of communication is described in ICF mainly as ability to understand the various spoken, non verbal, body messages. Thus, we mapped it to the SUMO *Interpreting *class, which is defined as the process of assigning a *Proposition *to a *Text*. However, it would be better mapped to a superclass of *Interpreting*, where the process of understanding is applied to a more generic *ContentBearingObject*.

Chapter 4 is about mobility, of oneself as well as of objects. The SUMO class that seems to capture the meaning of this chapter is *Motion*. However, *Motion *in SUMO is not an *IntentionalProcess*.

Chapters 5 (Self care) and 6 (Domestic life) are subclasses of *IntentionalProcess*, with some but not all specific concepts being available also in SUMO (e.g., *d540 Dressing*, *d550 Eating*, *d560 Drinking*, etc.).

Chapter 7 relates to interpersonal interactions and relationships, their properties, their timeline, their formality or informality, and other aspects. As such, no direct mapping can be found for most concepts.

When looking at the other chapters that surely describe participation (like 8 and 9), some concepts can be mapped to SUMO *SocialInteraction*, and others to more general processes.

## Conclusions

Our analysis of the relationships within ICF identified some issues, confirming what already observed by Kumar and Smith on a different component [7], and in particular problems related to confusion between classes and their properties, incorrect classifications, and overemphasis on subsumption.

The formal problems described here may not be considered as limits of ICF, as well as of other traditional biomedical terminologies and classifications (see, e.g., [15] for SNOMED), but they can be also considered unique opportunities for an ontological enhancement of ICF as envisaged by WHO.

The main finding is that ICF activities mostly correspond to SUMO intentional processes, although their participation facet has not been mapped to any SUMO concept. In addition to that, two chapters (d2 and d7) represent a metaclassification that is not yet fully exploited inside ICF.

To proceed towards a more formal representation of ICF, a model could be developed based on properties described in formal ontologies for the mapped classes, and adding what needed to represent the specific ICF knowledge on activities and participation. According to our findings, interesting properties for activities might include the number of agents involved, the instrument used to carry out the activity and if necessary the object (patient) of the activity, some measures of the complexity of the task, and possibly an enumeration of relevant subtasks. Although in this exploration we did not consider qualifiers role, the proposed analysis may help in driving future evolutions of ICF towards a more formally founded description of human activities.

## Abbreviations

ClaML: Classification Markup Language; ICD: International Classification of Diseases; ICF: International Classification of Functioning, Disability and Health; SNOMED: Systematized Nomenclature of Medicine; SUMO: Suggested Upper Merged Ontology.

## Competing interests

The authors declare that they have no competing interests.

## Authors' contributions

VDM conceived the study, collaborated in the analysis and drafted the paper. AS participated in the analysis and collaborated in the writing of the final version of the paper. Both authors have read and approved the manuscript.

## Supplementary Material

Additional file 1 **Graphical representation of the relationships in ICF VDM-AP-map.pdf graphically describes the relationships found in the analysis of the Activities and Participation component of ICF**. Click here for file
