# Supplementary figures and images for: An ontology-based exploration of the concepts and relationships in the activities and participation component of the international classification of functioning, disability and health
Source: J Biomed Semantics. 2012 Feb 28;3:1. doi: 10.1186/2041-1480-3-1 (PMC3310852; doi:10.1186/2041-1480-3-1)

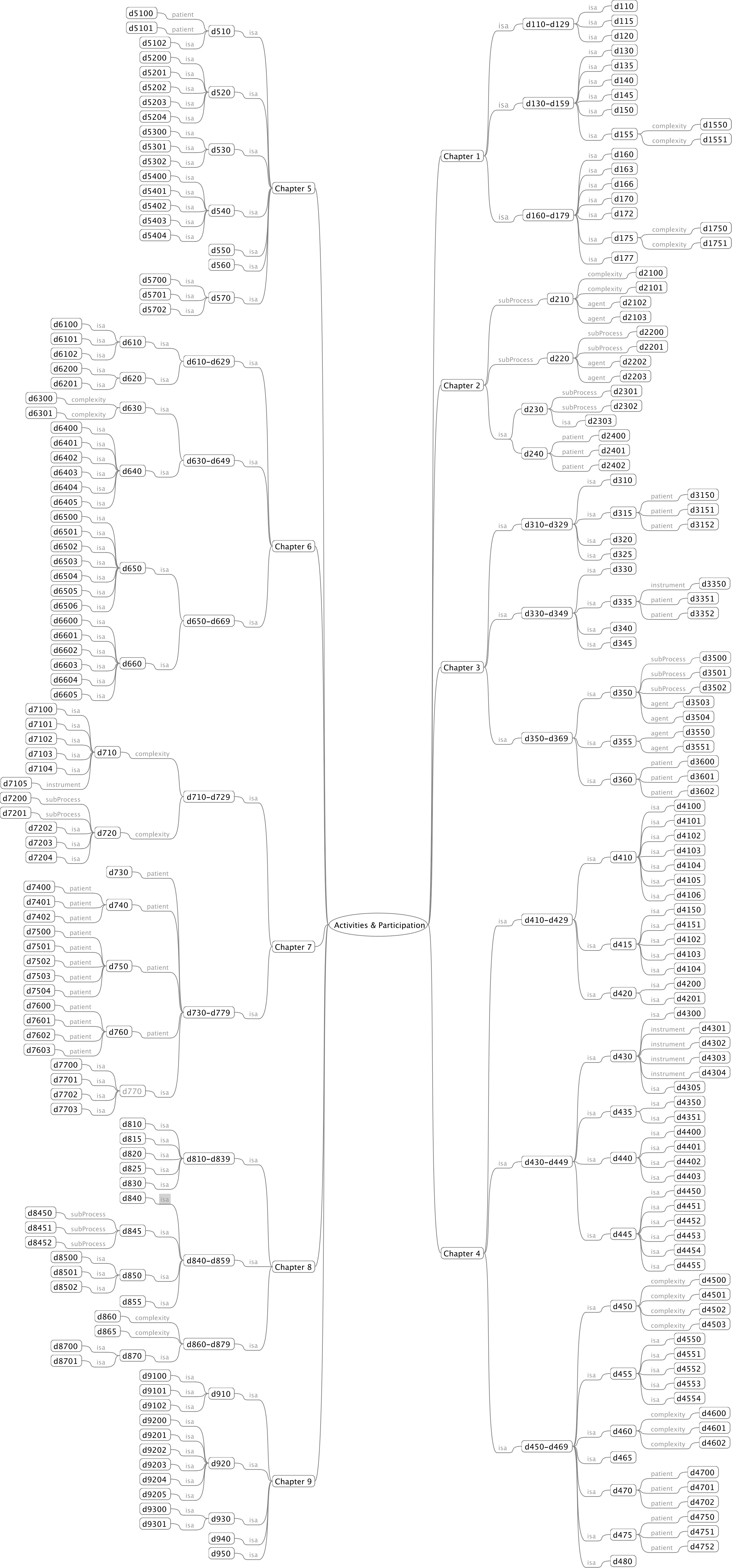

Supplement: Additional file 1 — Graphical representation of the relationships in ICF VDM-AP-map.pdf graphically describes the relationships found in the analysis of the Activities and Participation component of ICF. [file 2041-1480-3-1-S1.PDF]
